# Antibacterial activity of the novel compound Sudapyridine (WX-081) against *Mycobacterium abscessus*

**DOI:** 10.3389/fcimb.2023.1217975

**Published:** 2023-08-17

**Authors:** Wenjuan Nie, Shan Gao, Lei Su, Lina Liu, Ruixue Geng, Yingxia You, Naihui Chu

**Affiliations:** ^1^ Tuberculosis Department, Beijing Chest Hospital, Capital Medical University, Beijing, China; ^2^ Tuberculosis Department, Beijing Tuberculosis and Thoracic Tumor Research Institute, Beijing, China; ^3^ Tuberculosis Department, Henan Anyang City Tuberculosis Prevention and Control Institute, Anyang, China; ^4^ Tuberculosis Department, Hengshui Third People’s Hospital, Hengshui, China; ^5^ Tuberculosis Department, Hohhot Second Hospital, Hohhot, China; ^6^ Tuberculosis Department, Zhengzhou Sixth People’s Hospital, Zhengzhou, China

**Keywords:** *Mycobacterium abscessus*, Sudapyridine, zebrafish, activity, *in vivo*

## Abstract

**Objective:**

This study aimed to investigate sudapyridine (WX-081) antibacterial activity against *Mycobacterium abscessus in vitro* and its effect on *in vivo* bacterial growth and host survival using a zebrafish model of *M. abscessus* infection.

**Methods:**

WX-081 *in vitro* antibacterial activity was assessed based on growth inhibition of *M. abscessus* standard strain ATCC19977 and 36 clinical isolates. Maximum tolerated concentrations (MTCs) of WX-081, bedaquiline, and azithromycin and inhibition of *M. abscessus* growth were assessed *in vivo* after fluorescently labelled bacilli and drugs were injected into zebrafish. Bacterial counts were analysed using one-way ANOVA and fluorescence intensities of zebrafish tissues were analysed and expressed as the mean ± SE. Moreover, Kaplan-Meier survival analysis was conducted to assess intergroup differences in survival of *M. abscessus*-infected zebrafish treated with different drug concentrations using a log-rank test, with a p value <0.05 indicating a difference was statistically significant.

**Results:**

Drug sensitivity testing of *M. abscessus* standard strain ATCC19977 and 36 clinical isolates revealed MICs ranging from 0.12-0.96 µg/mL and MIC_50_ and MIC_90_ values of 0.48 µg/mL and 0.96 µg/mL, respectively. Fluorescence intensities of *M. abscessus*-infected zebrafish tissues was lower after treatment with the WX-081 MTC (62.5 µg/mL) than after treatment with the azithromycin MTC (62.5 µg/mL) and the bedaquiline MTC (15.6 µg/mL). When the concentration of WX-081 increased from 1.95µg/mL to 1/8 MTC(7.81µg/mL), the survival rate of zebrafish at 4-9 dpf decreased from 90.00% to 81.67%.

**Conclusion:**

WX-081 effectively inhibited *M. abscessus* growth *in vitro* and *in vivo* and prolonged survival of *M. abscessus*-infected zebrafish, thus indicating that WX-081 holds promise as a clinical treatment for *M. abscessus* infection.

## 1 Introduction

Incidence and associated mortality rates of lung infections caused by nontuberculous mycobacteria (NTM) are increasing worldwide, due to obstacles that are impeding effective management of NTM disease (Cowman et al., 2019). Importantly, NTM is a heterogeneous group that includes fast-growing and slow-growing species, with *Mycobacterium abscessus* the most commonly detected fast-growing mycobacterial species in patients with NTM infections. Multi-drug regimens containing macrolides are strongly recommended for the treatment of *M. abscessus* lung disease, as indicated in NTM therapeutic treatment guidelines that were jointly issued by The American Thoracic Society (ATS), European Respiratory Society (ERS), European Society of Clinical Microbiology and Infectious Diseases (ESCMID) and the Infectious Diseases Society of America (IDSA) (Daley et al., 2020). Moreover, the studies have shown that azithromycin(AZM) has more antibacterial effect on Mycobacterium abscess both *in vivo* and *in vitro*, and it is not easy to develop resistance to macrolides (Choi et al., 2012; Nie et al., 2014). Nevertheless, the choice of effective therapeutic agents for treating patients with *M. abscessus* lung infections is limited by the natural resistance of *M. abscessus* to commonly used antibiotics (Nessar et al., 2012).

Bedaquiline (BDQ) is a diarylquinoline anti-tuberculosis antibiotic that inhibits mycobacterial adenosine triphosphate (ATP) synthase activity to thereby prevent ATP production that is required for numerous cellular activities (Andries et al., 2005). The World Health Organisation (WHO) advocates the use of BDQ as an initial oral treatment in combination with levofloxacin/moxifloxacin and linezolid to treat multidrug-resistant tuberculosis (MDR-TB) cases (World Health Organization, 2019). Importantly, BDQ is therapeutically effective against both MDR-TB and NTM (Pym et al., 2016; Ruth et al., 2019) and thus has potential value as a treatment for infections caused by *M. abscessus* complex species (Brown-Elliott and Wallace, 2019). However, BDQ-triggered adverse reactions, especially cardiac QT interval prolongation (Gao et al., 2021), have limited BDQ clinical applicability and prompted researchers to search for safer drugs for mycobacterial disease treatment. Sudapyridine (WX-081), a new diarylpyridine analogue (Huang et al., 2022), has similar anti-mycobacterial activity to that of BDQ, but is safer than BDQ (Yao et al., 2022). In fact, WX-081 is currently under evaluation in Phase III clinical trials in China as a tuberculosis treatment (JYP0081M301). Furthermore, preliminary results obtained in a previous study suggest that WX-081 has good anti-*M. abscessus* activity, prompting this study.

In this study, the antibacterial activity of WX-081 against *M. abscessus* was assessed *in vitro* using a standard *M. abscessus* strain. In addition, effects of WX-081 on *in vivo* bacterial growth and host survival were assessed using *M. abscessus*-infected zebrafish as a model of *M. abscessus* infection.

## 2 Materials and methods

### 2.1 Minimum inhibitory concentration determinations

AZM and BDQ are both purchased by Beijing Solarbio Science & Technology Co., Ltd.(Beijing, China), and WX-081 is provided by Shanghai Jiatan Biotechnology Co., Ltd. (Shanghai, China). Both drugs were dissolved in dimethyl sulfoxide (DMSO), and the drug solution was prepared according to the suggestions provided by the Institute of Clinical and Laboratory Standards (CLSI) (Woods et al., 2011). The standard strain of *M. abscessus* ATCC 19977, and 36 clinical strains of *M. abscessus* were cultured on solid lwenstein-Jensen medium at 37°C for 4-6 days. The MICs of AZM, BDQ and WX-081 were determined by adding drugs to *M. abscessus* cultured in 96-well plates according to the recommended CLSI broth microdilution method. The broth was diluted twice, and the concentrations of AZM ranged from 0.5-256μg/mL, both BDQ and WX-081 ranged from 0.015-7.68μg/mL. A bacterial inoculum with turbidity equivalent to 0.5 McFarland standard dilution of 1∶200 was prepared for each strain. The MIC of *M. abscessus* was determined after 3 days of culture at 37°C together with antibiotics. Thereafter, 70 μl of Alamar Blue solution (Sirotec, 20μL Alamar Blue+50μL 5% Tween 80 of 5% Tween 80) was added to each well, and then the plates were incubated for another 24 hours. The color change from blue to pink indicates bacterial growth (Cowman et al., 2016). MIC is defined as the lowest drug concentration that no color change, that is, the lowest concentration that can inhibit the visible growth of the test isolate. Explain the results of drug sensitivity test (DST) according to the breakpoint recommended by CLSI.

For species with enough isolates and for which WX-081 demonstrated good inhibitory activity, The epidemiological cut-off (ECOFF) was determined according to the distribution profile of the MIC values. ECOFF is defined as the concentration that can inhibit > 95% of the bacterial population for the unimodal MIC distribution curve. For the bimodal MIC distribution curve, ECOFF is set between the two peaks.

### 2.2 Microinjection of *M. abscessus* into zebrafish as an *in vivo M. abscessus* infection model

This study was approved by the Ethics Committee of Beijing Chest Hospital, Capital Medical University(2021-020). The wild-type AB zebrafish strain selected for the study was propagated via natural paired mating and reproduction and maintained in unchlorinated fresh water at 28°C and fed commercial fish food. *M. abscessus* ATCC19977 bacilli with smooth (S) morphology were incubated for 5 to 7 days at 30°C in Middlebrook 7H9 broth (Becton Dickinson) containing 10% OADC (Becton Dickinson) and 0.05% Tween 80 (Sigma-Aldrich). When cultures reached mid-log-phase growth, they were centrifuged then pellets were washed and resuspended in phosphate-buffered saline (PBS) containing 0.05% Tween 80. Next, the bacterial suspensions were homogenised and sonicated then the tubes were allowed to stand without shaking to allow bacteria to settle to the bottoms of tubes for 5 to 10 min. After the liquid in tubes was carefully removed, the settled bacterial cells were collected and resuspended in PBS then DiO green fluorescent dye was added to the cell suspensions to fluorescently label the bacilli. Thereafter, the bacteria were transplanted into zebrafish of the wild-type AB strain at 2 days post-fertilisation (dpf) via micro-venous injection. Zebrafish were anesthetized using 3-aminobenzoic acid ethyl ester methanesulfonate (C_9_H_11_NO_2_·CH_4_O_3_S, MESAB). The MESAB was prepared by mixing MESAB and Na_2_HPO_4_·12H_2_O in a total mass ratio of 1:5 to make a 4 mg/mL solution, which was stored at 4°C. For use, it was diluted with standard dilution water, with a final anesthetic concentration of 0.16 mg/mL. Then the zebrafish was injected bacteria by intravenous microinjection with each fish receiving approximately 3.6×10³ colony forming units (CFUs) of the transplant to establish the zebrafish model of *M. abscessus* infection (Guo et al., 2020; Nie et al., 2020).

### 2.3 Maximum tolerated concentrations of AZM, BDQ, and WX-081 in zebrafish

Zebrafish at 3 dpf were viewed under a microscope then they were randomly allocated to six-well plates (30 zebrafish per well) in 3 mL of water/well and maintained at 35°C throughout the experiment. AZM, BDQ, and WX-081 stock solutions were each prepared in 20.0 mg/mL DMSO solutionand stored at −20°C until needed. For experiments, drugs were diluted in water to generate 2-fold serial dilutions of AZM (62.5 μg/mL, 125 μg/mL, 250 μg/mL, 500 μg/mL, and 1000 μg/mL), BDQ (3.91 μg/mL, 7.81 μg/mL, 15.6 μg/mL, 31.2 μg/mL, and 62.5 μg/mL), and WX-081 (15.7 μg/mL, 31.3 μg/mL, 62.5 μg/mL, 125 μg/mL, 250 μg/mL, 500 μg/mL, and 1000 μg/mL). Zebrafish of drug-treated groups (designated AZM group, BDQ group, and WX-ZM group) received *M. abscessus* injections followed by additions of diluted drug treatments added directly to plate wells. Concurrently, blank control group and model group zebrafish were maintained using the same protocol, but the blank control group received no *M. abscessus* injection and no drug treatment, while model group zebrafish each received one *M. abscessus* injection and no drug treatment. After zebrafish were treated with control (no drug) or experimental (drug) treatments for 48 h at 35°C, MTCs of control groups (blank control, model) and experimental groups (AZM, BDQ, and WX-081 groups) were determined.

### 2.4 Evaluation of the *in vivo* inhibitory effects of WX-081 on bacterial growth and host survival using the *M. abscessus*-infected zebrafish model

According to previously reported procedures, zebrafish at 3 dpf were viewed under a microscope then randomly assigned to 6-well plates (30 zebrafish/well). Drug stocks were diluted with water to create diluted AZM, BDQ, and WX-081 solutions in 3-mL volumes at concentrations of 62.5 μg/mL for AZM, 15.6 μg/mL for BDQ, and 1.95 μg/mL, 3.91 μg/mL, 7.81 μg/mL, 15.6 μg/mL, 31.2 μg/mL, and 62.5 μg/mL for WX-081. Control group and model group samples were set up at the same time in 3 mL of water per well. Live zebrafish was used for imaging. The zebrafish was anesthetized and transferred onto methyl cellulose using a Pasteur pipette. An electrically controlled, continuously variable magnification fluorescence microscope (AZ100, Nikon, Japan) equipped with a green fluorescence channel was used at a magnification of 30x. After 48 h of drug treatment at 35°C, 10 randomly selected zebrafish in each experimental group were digitally photographed under a fluorescent microscope. Image processing software (NIS-Elements D 3.20) was used to collect and analyse image-based data to determine drug efficacies for inhibiting the *M. abscessus* growth *in vivo* based on zebrafish whole-body fluorescence intensity data.

### 2.5 Evaluation of WX-081 efficacy in prolonging survival time of zebrafish infected with *M. abscessus*

In order to evaluate whether AZM, BDQ, and WX-081 can prolong the survival time of M. abscessus-infected zebrafish and the difference in the prolonged time between them, the number of zebrafish surviving and dying in each experimental group should be observed and recorded every day, and the dead individuals should be removed in time until 9 dpf. Zebrafish at 3 dpf were viewed under the microscope then were randomly transferred to 50-mL beakers (experimental group) (60 zebrafish/beaker). AZM, BDQ, and WX-081 were diluted to generate working drug solutions of AZM (62.5 μg/mL), BDQ (15.6 μg/mL), and WX-081 (1.95 μg/mL, 3.91 μg/mL, and 7.81 μg/mL) in a 20-mL volume/beaker. Control and model groups were set up at the same time in a 20-mL volume per beaker. All groups were incubated at 35°Cduring the treatment period (48 h). Each day, numbers of dead zebrafish were recorded and dead zebrafish were removed. Survival analysis was performed on the survival time and survival quantity.

### 2.6 Data analysis

Data were statistically analysed using SPSS 26.0. Fluorescence intensities were expressed as the mean ± SE. Student’s t or t’ tests for independent samples were used for comparisons between two groups if data were normally distributed; non-parametric tests were used if data were non-normally distributed. Kaplan-Meier survival analysis was performed using the log-rank test to determine survival rates of WX-081-treated zebrafish at different drug concentrations. CFU counts obtained for different groups were analysed and compared using one-way ANOVA, with a p value of <0.05 indicating statistically significant intergroup differences were found.

## 3 Results

### 3.1 MIC assay of WX-081 against *M. abscessus*

**Figure 1:**
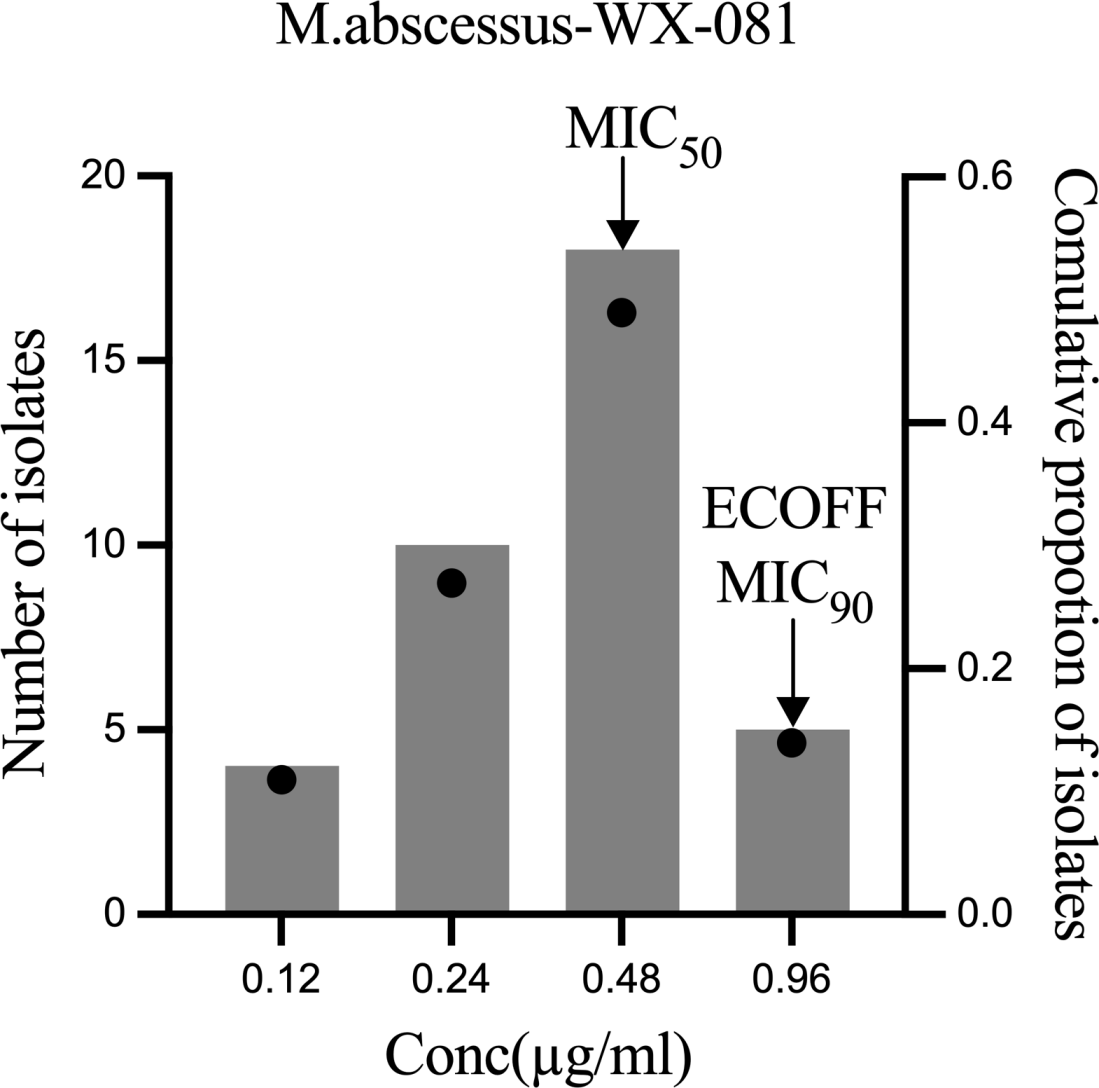
WX-081 MIC distribution for M. abscessus. Black dots represent the cumulative proportion of strains with each MIC value. The bars represent the number of isolates corresponding to each MIC value.

MICs of WX-081 against *M. abscessus* ATCC19977, and 36 clinical isolates ranged from 0.12 μg/mL to 0.96 μg/mL, with a MIC_50_ of 0.48 µg/mL and a MIC_90_ of 0.96 µg/mL observed (**Figure 1**). Ultimately, WX-081 exerted strong antibacterial activity against almost all tested *M. abscessus* isolates, as evidenced by MICs that were generally much lower than 1 µg/mL. The ECOFF value of WX-081 against *M. abscessus* was 0.96 µg/mL. The MIC of *M. abscessus* ATCC19977, MIC range, MIC_50_ and MIC_90_ of AZM, BDQ and WX-081 is showed in **Table 1**.

**Table 1:** MIC of AZM, BDQ and WX-081 against M. abscessus.

Drug	MIC of ATCC19977(µg/mL)	MIC Range(µg/mL)	MIC_50_(µg/mL)	MIC_90_(µg/mL)
AZM	0.5	0.5-128	2	8
BDQ	0.12	0.12-1.92	0.12	0.96
WX-081	0.12	0.12-0.96	0.48	0.96

### 3.2 AZM, BDQ, and different concentrations of WX-081 in zebrafish MTC *in vivo*

**Table 2** shows MTC values for AZM, BDQ, and WX-081 groups of 62.5 µg/mL, 15.6 µg/mL, and 62.5 µg/mL, respectively. Notably, when AZM was administered at 125 μg/mL, zebrafish health began to deteriorate; at 500 μg/mL, blood stasis in the heart was observed; at 1000 μg/mL, the zebrafish mortality rate reached 33.33%. When the concentration of BDQ was increased from 3.91 μg/mL to 15.6 μg/mL, no obvious change was observed relative to the model group; at 31.2 μg/mL, four zebrafish had renal edema, two had pericardial edema, and one had blood stasis; at 62.5 μg/mL, only one zebrafish survived. When the concentration of WX-081 was 62.5 μg/mL (the MTC), zebrafish were healthy; when the concentration was increased to 125 μg/mL, zebrafish exhibited signs of heart congestion and their bodies became bent, although no deaths of zebrafish were observed; when the WX-081 concentration was increased from 250 μg/mL to 500 μg/mL, the zebrafish mortality rate increased from 10.00% to 100.00%.

**Table 2:** The MTC of AZM, BDQ and WX-081 in zebrafish *in vivo*.

Groups	concentration (μg/mL)	number of deaths (n)	mortality rate (%)	phenotype
Control group (n = 30)	–	0	0	normal
Model group (n = 30)	–	0	0	normal
AZM (n = 30)	62.5	0	0	similar to Model group
	125	0	0	5 body bent
	250	1	3.33	3 body bent
	500	1	3.33	1 heart congestion, 5 body bent
	1000	10	33.33	–
BDQ (n = 30)	3.91	0	0	similar to Model group
	7.81	0	0	similar to Model group
	15.6	0	0	similar to Model group
	31.2	0	0	4 kidneys edema, 2 pericardial edema and 1 heart congestion
	62.5	29	6.67	–
WX-081 (n = 30)	15.7	0	0	similar to Model group
	31.3	0	0	similar to Model group
	62.5	0	0	similar to Model group
	125	0	0	2 heart congestion, 3 body bending
	250	3	10.00	–
	500	30	100.00	–
	1000	30	100.00	–

When zebrafish mortality exceeded 10%, the toxic phenotype was not evaluated.

### 3.3 Evaluation of *in vivo* inhibitory effects of AZM and BDQ and different concentrations of WX-081 on *M. abscessus* growth using the zebrafish model

AZM, BDQ, and WX-081 MTCs and WX-081 serial 2-fold MTC dilutions (1/32, 1/16, 1/8, 1/4, 1/2) are shown below with corresponding zebrafish whole-body and head fluorescence intensity values (**Table 3**). Pairwise comparisons of zebrafish whole-body fluorescence intensity values revealed lower values for AZM and BDQ groups vs. that of the model group (433684 ± 11910 vs. 671089 ± 22305, respectively, p < 0.001; 438648 ± 8280 vs. 671089 ± 22305, respectively, p < 0.001), with similar zebrafish head fluorescence intensity trends observed (78397 ± 4815 vs. 132412 ± 9989, p < 0.001; 79664 ± 5809 vs. 132412 ± 9989, p < 0.001, respectively).

**Table 3:** The efficacy of AZM, BDQ, and WX-081 against Mycobacterium Abscess infection in zebrafish *in vivo*.

Groups	concentration (μg/mL)	Whole-body fluorescence intensity (pixel, mean ± SE)	Head fluorescence intensity (pixel, mean ± SE)
Control group (n = 10)	–	361707 ± 7139**	55473 ± 2670**
Model group (n = 10)	–	671089 ± 22305	132412 ± 9989
AZM (n = 10)	62.5	433684 ± 11910**^a3^	78397 ± 4815**
BDQ (n = 10)	15.6	438648 ± 8280**^a1^	79664 ± 5809**
WX-081 (n = 10)	1.95	535952 ± 23902**^a1^	101579 ± 6065^a3^
	3.91	520519 ± 18208**^a1^	101495 ± 10160^a2^
	7.81	492988 ± 17182**^a1^	93190 ± 5468^a2^
	15.6	462296 ± 14360**^a1^	89877 ± 10333*
	31.2	419947 ± 13302**^a2^	76778 ± 4906**
	62.5	375347 ± 11359**	70976 ± 5726**

Compared with the model control group, *p < 0.05, **p < 0.01.

Compared with 62.5 μg/mL WX-081, ^a1^p < 0.001, ^a2^p < 0.05, ^a3^p < 0.01.

**Figure 2:**
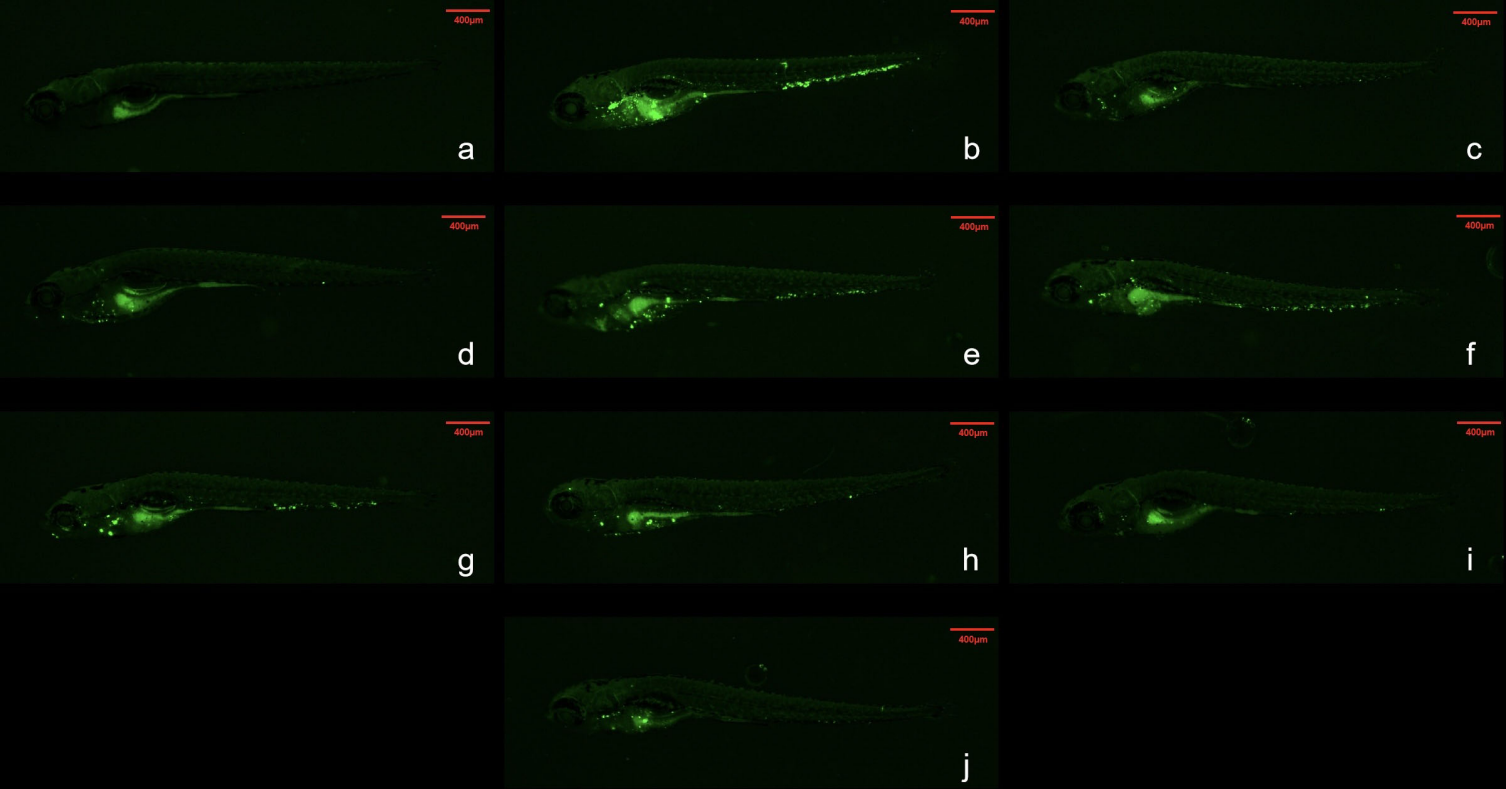
Fluorescence intensity distribution of different drugs in zebrafish. Green spots are Mycobacterium abscessus. The red dotted box represents the head analysis area. **(A)** Control group; **(B)** Model group; **(C)** 62.5μg/mL Azithromycin; **(D)** 15.6μg/mL Bedaquiline; **(E)** 1.95 μg/mL- WX-081; **(F)** 3.91 μg/mL-WX-081; **(G)** 7.81μg/mL-WX-081; **(H)** 15.6 μg/mL- WX-081; **(I)** 31.2 μg/mL- WX-081; **(J)** 62.5 μg/mL- WX-081.

**Figure 3:**
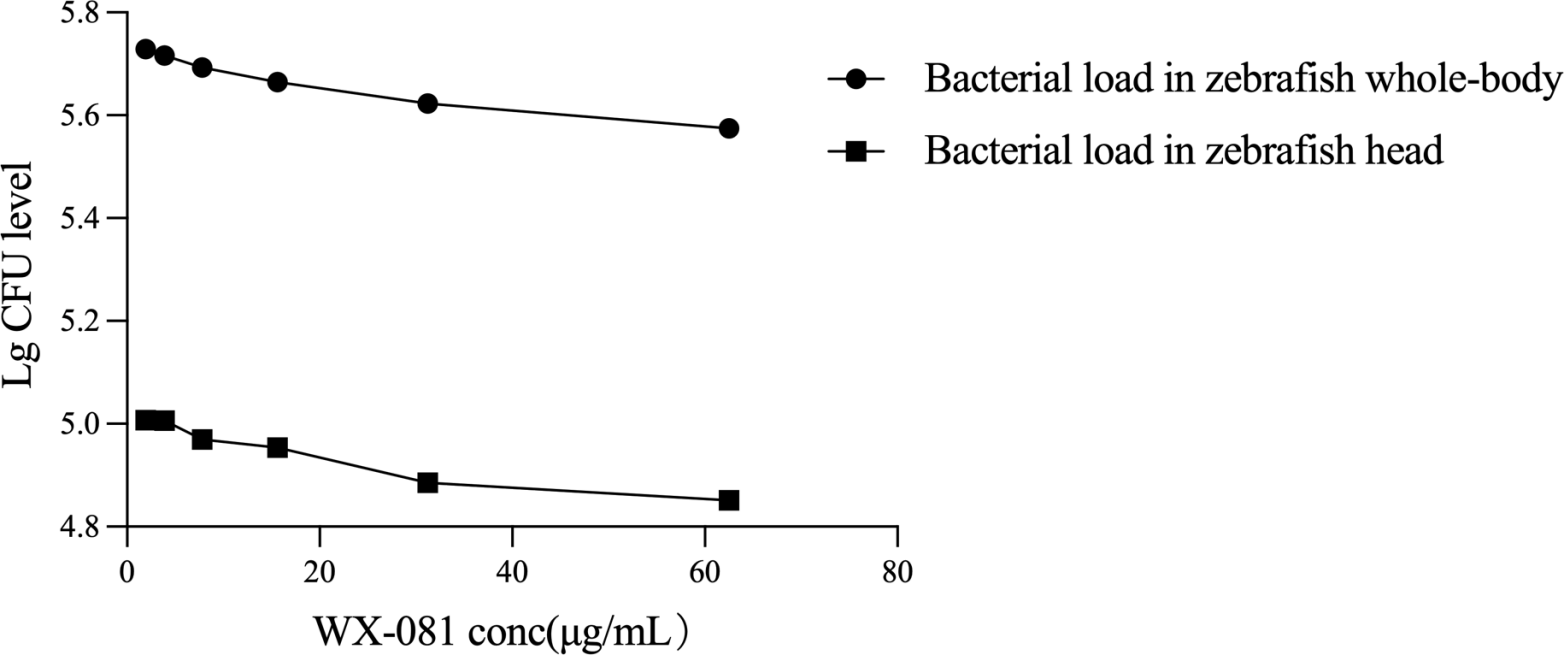
Therapeutic effect of different concentrations of WX-081 on M. abscessus infection in zebrafish whole-body and head.

An analysis of whole-body fluorescence intensities revealed a lower fluorescence intensity at the WX-081 MTC than at 1/32, 1/16, 1/8, 1/4, and 1/2 dilutions of the MTC. Fluorescence intensities of groups treated with the AZM MTC (62.5 μg/mL) and BDQ MTC (15.6 μg/mL) were each higher than that of the WX-081 MTC group (433684 ± 11910 vs. 375347 ± 11359, p < 0.01: 438648 ± 8280 vs. 375347 ± 11359, p< 0.001, respectively. By contrast, analysis of head fluorescence intensities revealed a lower fluorescence intensity at the WX-081 MTC than at 1/32, 1/16, and 1/8 dilutions of the WX-081 MTC that did not differ statistically from fluorescence intensities of AZM and BDQ MTC groups (78397 ± 4815 vs. 70976 ± 5726, respectively, p > 0.05. 79664 ± 5809 vs. 70976 ± 5726, respectively, p > 0.05). **Figure 2** shows *in vivo* fluorescence intensity distributions for zebrafish treated with different drugs. Increased WX-081 concentration was associated with lower bacterial burdens in both whole-body and head of zebrafish as determined by bacterial CFU (**Figure 3**).

### 3.4 Evaluation of WX-081 efficacy in prolonging survival of zebrafish infected with *M. abscessus*

**Figure 4:**
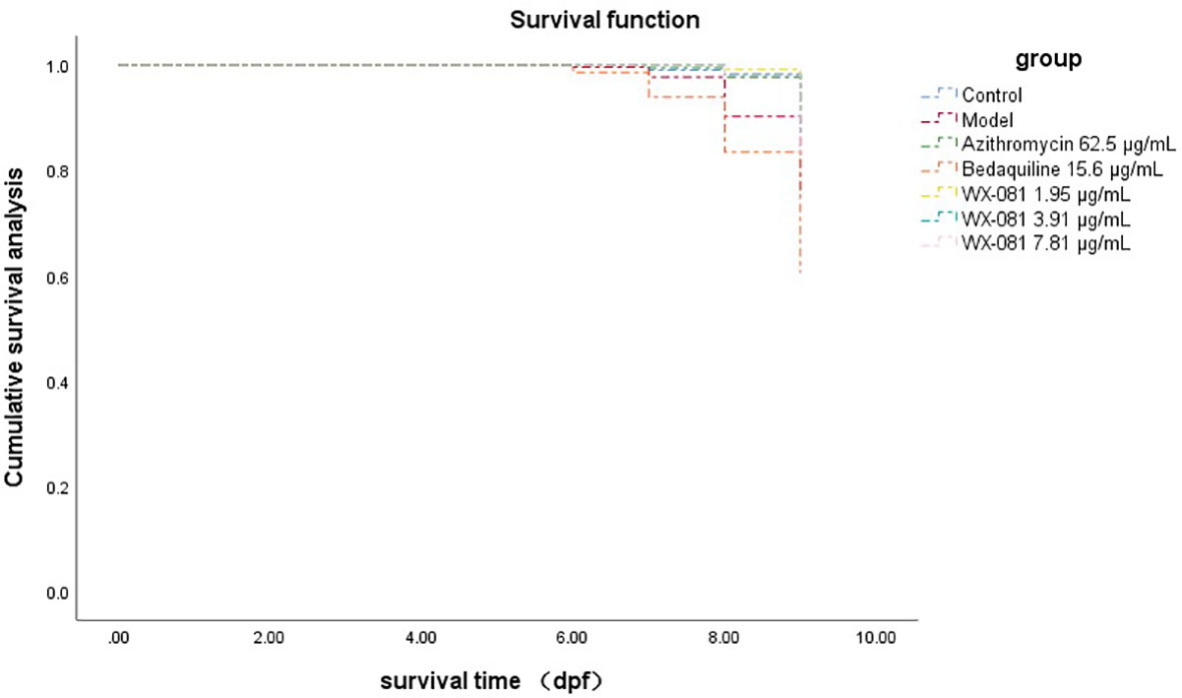
Kaplan-Meier survival curves for zebrafish with different drugs.

The survival rate of zebrafish in AZM MTC group at 4-9dpf was 88.33% higher than model group (Untreated *M. Abscessus*-Infected ZebraFish) 66.67% (p<0.01), however, the survival rate in the BDQ MTC group was lower than model group (48.33% vs.66.67%, p<0.05). When the concentration of WX-081 increased from 1.95μg/mL to 7.81μg/mL, the survival rate decreased from 90.00% to 81.67%, but it was still higher than that of the model group (**Table 4**). **Figure 4** shows survival curves of zebrafish treated with different concentrations of AZM, BDQ, and WX-081. The survival experiment was conducted after confirming the effectiveness of the antibacterial experiment. the highest concentration in the survival experiment was determined based on the MTC observed in zebrafish treated for 48 hours. Although the deaths caused by high drug concentrations were indeed due to the drug itself, the results at medium and low concentrations adapted for demonstrating the ability of the drug to inhibit bacterial growth and extend the survival time of zebrafish.

**Table 4:** Effect of AZM, BDQ and different concentrations of WX-081 of survival in zebrafish.

Groups	concentration(μg/mL)	4-9 dpfsurvival rate(%)
Control group (n = 60)	–	91.67^b3^
Model group (n = 60)	–	66.67
AZM (n = 60)	62.5	88.33^b2^
BDQ (n = 60)	15.6	48.33^b1^
WX-081 (n = 60)	1.95	90.00^b2^
	3.91	85.00^b1^
	7.81	81.67^b1^

Compared with the model control group, ^b1^p < 0.05,^b2^p < 0.01,^b3^p < 0.001.

## 4 Discussion

Macrolide drugs are the basis of multidrug treatment of M. abscessus infection, but the success rate of treatment is very low, especially for macrolide drug-resistant strains (Richard et al., 2020). The potent efficacy of BDQ treatment for MDR-TB is well recognised, however, the cardiotoxicity of this drug poses a significant risk to patient health and thus has limited its use for TB treatment. To address this issue, a structural BDQ analogue with comparable bacteriostatic efficacy and better safety is needed. One such BDQ analogue, WX-081, has recently been reported in an animal model-based study to exert comparable antibacterial activity to that exerted by BDQ, besides, the quantitative ECG changes occurred in animals after BDQ application, which may be related to cardiotoxicity of BDQ, but no relative changes occurred after WX-081 application (Yao et al., 2022). Nevertheless, although WX-081 is an innovative new drug that is currently under evaluation as a treatment for MDR-TB by the Chinese National Medical Products Administration (NMPA), many key activities of this drug have not yet been reported. Results obtained in one study showed that WX-081 levels exceeded corresponding BDQ levels in mouse and rat tissues; this effect was especially pronounced in the lung, whereby at 96 h after dosing, WX-081 levels were several times greater than BDQ levels (Yao et al., 2022). Meanwhile, results of another study (Zhu et al., 2022) evaluating WX-081 anti-mycobacterial activity revealed that *M. abscessus* strains were susceptible to the drug, although only a small number of strains were evaluated, warranting further study. The study (Dupont et al., 2017) has shown that BDQ is very effective against *M. abscessus* in zebrafish model, and short-term treatment with BDQ is enough to produce protective effect. Besides, the previous study (Nie et al., 2014) have shown that the main pathogenic subspecies isolated from human pathogenic *M. abscessus* strains are S-type/smooth type, not R-type/rough type, so it is more important to analyze S-type.

Zebrafish are small (< 4-cm-long) freshwater fish that are used as animal models of human diseases, due to similarities of their innate and acquired immune systems to corresponding mammalian systems. This advantage has prompted some researchers to adopt zebrafish-based models over invertebrate models (e.g., *Drosophila melanogaster*, nematodes) (van der Sar et al., 2004). Other advantages of zebrafish models include gene screening and real-time visualisation features not provided by other vertebrate models. Consequently, in recent years increasing numbers of researchers have used the zebrafish model of *M. abscessus* infection to investigate *in vivo* antibacterial activities of drugs (Bernut et al., 2014; Lefebvre et al., 2017) (Dupont et al., 2017; Winters et al., 2022).

In this investigation of bacteriostatic activities of WX-081 against various *M. abscessus* isolates, MICs WX-081 against *M. abscessus* ranged from 0.12 µg/mL to 0.96 µg/mL, thus demonstrating thus demonstrating the good inhibition activity for *M. abscessus in vitro*. In addition, fluorescence intensities of fluorescently-labelled *M. abscessus* in zebrafish tissues gradually decreased as the WX-081 concentration was increased from 1.95 μg/mL to 7.81 μg/mL and the survival rate of zebrafish at 4-9 dpf decreased from 90.00% to 81.67%, thus suggesting that WX-081 also may provide good inhibition activity for *M. abscessus in vivo*.

Due to the fact that WX-081 is a new antibiotic, here we report for the first time the WX-081 *in vivo* bacteriostatic activity, as based on its effect on survival of *M. abscessus*-infected zebrafish. To date, only one *in vitro* study of the antibacterial activity of WX-081 against *M. abscessus* has been reported (Zhu et al., 2022), which revealed a WX-081 MIC of 0.25 μg/mL. In the current study, whole-body fluorescence intensity of zebrafish infected with fluorescently labelled *M. abscessus* after treatment with the WX-081 MTC (62.5 µg/mL) was lower than that observed for other WX-081 concentrations. Moreover, the survival rate of zebrafish at 4-9 dpf decreased from 90.00% to 81.67% as the concentration of WX-081 was increased from 1.95 μg/mL to the 1/8MTC. Taken together the abovementioned results conducted in a zebrafish model of *M. abscessus* infection indicated that WX-081 may inhibit *M. abscessus* growth *in vivo*. In addition, in this study of the bacteriostatic activity of WX-081 *in vitro*, the MIC of WX-081 was about equal to or twice of MIC of BDQ, similar results were also found in the study of the comparison in inhibition mycobacterium tuberculosis effects between WX-081 and BDQ (Xiao et al., 2023).

In this study, we investigated WX-081 effects on *M. abscessus* within the zebrafish head, whereby analysis of head fluorescence intensity revealed lower intensity at the WX-081 MTC as compared to that of the model group (p < 0.001) and to those of groups treated with 1/32 (p < 0.01), 1/16 (p < 0.05), 1/8 (p < 0.05) of the WX-081 MTC. As the drug concentration was gradually increased, the fluorescence intensity of the head gradually decreased and the bacterial count in the head decreased linearly with increasing drug concentration, thus suggesting that WX-081 effectively inhibited *M. abscessus* present within zebrafish heads. Similarly, results of a previous study had suggested that BDQ freely entered the cerebrospinal fluid of pulmonary TB patients with intact blood-brain barrier function. In this study, it was observed that with the increase of WX-081 concentration, the fluorescence intensity of zebrafish head decreased, indicating that WX-081 reduced the bacterial load in zebrafish head tissue, further demonstrating that the drug may have bacteriostatic effect on *M. abscessus* in zebrafish. Nonetheless, clinical studies are needed to determine whether WX-081 can meaningfully contribute to the treatment of central nervous system tuberculosis cases.

This study had several limitations. First, while conducting our *in vivo* experiments using the zebrafish model, we only analysed the bacteriostatic activity of WX-081 against a standard strain of *M. abscessus* and thus did not analyse its bacteriostatic activity against additional *M. abscessus* complex subspecies, including *M. abscessus* subsp. *massiliense* and *M. abscessus* subsp. *bolletii*. Second, the number of *M. abscessus* clinical isolates analysed in this study was limited and thus should be expanded in future studies to provide more reliable real-world data help clinicians select the most effective and safe drugs for their patients.

In conclusion, WX-081 can inhibit the growth of *M. abscessus* and prolong survival time of *M. abscessus*-infected zebrafish. Taken together, these results indicate that WX-081 is a promising new drug for use in treating *M. abscessus* infections.

## Data availability statement

The original contributions presented in the study are included in the article/supplementary material. Further inquiries can be directed to the corresponding author.

## Ethics statement

The animal study was reviewed and approved by the Ethics Committee of Beijing Chest Hospital, Capital Medical University

## Author contributions

WN and SG contributed to the experimental operation and article writing. LS, LL, RG, YY contributed to the experimental operation. NC contributed to the revision of the article. All authors contributed to the article and approved the submitted version.
